# Optimization of molecularly targeted MRI in the brain: empirical comparison of sequences and particles

**DOI:** 10.2147/IJN.S158071

**Published:** 2018-07-25

**Authors:** Niloufar Zarghami, Alexandre A Khrapitchev, Francisco Perez-Balderas, Manuel Sarmiento Soto, James R Larkin, Luca Bau, Nicola R Sibson

**Affiliations:** 1 Cancer Research UK and Medical Research Council Oxford Institute for Radiation Oncology, Department of Oncology, University of Oxford, Oxford, UK, nicola.sibson@oncology.ox.ac.uk; 2 Institute of Biomedical Engineering, Department of Engineering Sciences, University of Oxford, Oxford, UK

**Keywords:** molecular imaging, preclinical MRI, VCAM-MPIO, VCAM-USPIO, multi gradient echo, bSSFP

## Abstract

**Background:**

Molecular MRI is an evolving field of research with strong translational potential. Selection of the appropriate MRI sequence, field strength and contrast agent depend largely on the application. The primary aims of the current study were to: 1) assess the sensitivity of different MRI sequences for detection of iron oxide particles in mouse brain; 2) determine the effect of magnetic field strength on detection of iron oxide particles in vivo; and 3) compare the sensitivity of targeted microparticles of iron oxide (MPIO) or ultra-small superparamagnetic iron oxide (USPIO) for detection of vascular cell adhesion molecule-1 (VCAM-1) in vivo.

**Methods:**

Mice were injected intrastriatally with interleukin 1β to induce VCAM-1 expression on the cerebral vasculature. Subsequently, animals were injected intravenously with either VCAM-MPIO or VCAM-USPIO and imaged 1 or 13 hours post-injection, respectively. MRI was performed at 4.7, 7.0, or 9.4 T, using three different *T*_2_*-weighted sequences: single gradient echo 3D (GE3D), multi-gradient echo 3D (MGE3D) and balanced steady-state free precession 3D (bSSFP3D).

**Results:**

MGE3D yielded the highest signal-to-noise ratio (SNR) and contrast-to-noise ratio (CNR) for the detection of iron oxide particles. All sequences showed a significant increase in SNR and CNR from 4.7 to 7.0 T, but no further improvement at 9.4 T. However, whilst targeted MPIO enabled sensitive detection of VCAM-1 expression on the cerebral vasculature, the long half-life (16.5 h vs 1.2 min) and lower relaxivity per particle (1.29×10^–14^ vs 1.18×10^–9^ Hz L/particle) of USPIO vs. MPIO rendered them impractical for molecular MRI.

**Conclusion:**

These findings demonstrate clear advantages of MPIO compared to USPIO for molecularly-targeted MRI, and indicate that the MGE3D sequence is optimal for MPIO detection. Moreover, higher field strengths (7.0/9.4 T) showed enhanced sensitivity over lower field strengths (4.7 T). With the development of biodegradable MPIO, these agents hold promise for clinical translation.

## Introduction

Preclinical molecular and cellular MRI is an evolving field of research with strong translational potential. Iron oxide particles are currently the preferred contrast platform for this application and have been thoroughly reviewed in the literature.^1^–^4^ The magnetic core of these particles causes inhomogeneity in the local magnetic field, which leads to reduction of relaxation times of adjacent protons. Thus, the accumulation of iron oxide particles appears as dark (hypointense) regions on commonly used *T*_2_*/*T*_2_-weighted magnetic resonance images.^5^ The degree of hypointense image contrast, or the susceptibility effect, is highly dependent on relaxivity values and the iron concentration of these agents,^6^,^7^ as well as MRI parameters such as magnetic field strength^8^,^9^ and pulse sequence.^8^,^10^,^11^ However, although *T*_2_-weighted spin-echo (SE) and *T*_2_*-weighted gradient-echo (GRE) sequences are regarded as the gold standard for iron oxide-enhanced MRI,^12^–^14^ and 7.0 T is the most commonly used field strength preclinically, a robust comparison of other sequences and/or magnetic field strengths has yet to be demonstrated.

Iron oxide agents are usually composed of a superparamagnetic core and a shell of varying thickness and material. In general, these particles are classified into 3 categories based on their hydrodynamic size. Ultra-small superparamagnetic iron oxide (USPIO; ~5–50 nm in diameter) particles are the smallest agents currently in use.^7^,^15^ These particles typically exhibit a long (several hours) blood circulation half-life and have been clinically used for perfusion mapping and enhanced functional MRI activation detection.^16^,^17^ USPIO have also shown potential for imaging brain tumors with an impaired blood–brain barrier (BBB).^18^–^20^ Superparamagnetic iron oxide (SPIO; 50–150 nm in diameter) particles are slightly larger in size,^14^,^20^,^21^ and have seen extensive clinical use for lymph node imaging in cancer patients.^22^,^23^ Finally, microparticles of iron oxide (MPIO; ~1 μm^2^,^24^,^25^) have been used in a variety of preclinical molecular imaging,^25^–^29^ but until recently^30^ a clinically compatible MPIO platform has not existed. Innovations and developments in the field of molecular imaging and biodegradable iron oxide contrast agents have increased the potential for translation of preclinical research to clinic.^30^–^32^ However, selection of the appropriate USPIO, SPIO, or MPIO contrast agent for imaging depends largely on the application and research or clinical question to be addressed.

Over the last decade, molecular MRI, of brain–immune system interactions, has been the subject of numerous studies,^27^,^33^–^38^ with iron oxide particles being conjugated to a variety of different ligands (including proteins, peptides, antibodies, and carbohydrates) to target specific molecular epitopes expressed in disease. MPIO are often the agent of choice for such targeted molecular imaging studies, having several postulated advantages over UPSIO. The micron size of these particles enables assessment of specific binding to the endovasculature, unlike USPIO that are susceptible to macrophage uptake and passive diffusion into the brain parenchyma across a leaky BBB.^39^,^40^ Moreover, the iron content of a single MPIO is 4.3 million times higher than that of a USPIO, yielding considerably greater contrast per particle, with the contrast effect extending up to 50 times the physical size of the particle.^6^,^24^,^41^,^42^ Finally, the increased surface area of MPIO greatly increases labeling valency and, hence, binding affinity to the molecule of interest, while their short blood half-life (<5 min) compared to USPIO yields a high target-to-background ratio.^38^,^43^,^44^ However, many of these advantages remain theoretical or have only been demonstrated in vitro, and a direct head-to-head in vivo comparison of molecularly targeted MPIO and USPIO has not previously been reported.^37^,^39^

The primary aims of the current study, therefore, were to 1) assess the relative sensitivities of 3 commonly used MRI sequences (single gradient echo 3D, multi-gradient echo 3D, and balanced steady-state free precession 3D) for the detection of iron oxide particles in mouse brain; 2) determine the effect of magnetic field strength on the detection of iron oxide particles in vivo; and 3) compare the specificity and sensitivity of vascular cell adhesion molecule (VCAM)-MPIO and VCAM-USPIO for the detection of VCAM-1 in a mouse model of neuroinflammation.

## Materials and methods

### VCAM-MPIO synthesis

Pro-mag™ carboxylic acid MPIO^45^ (Cat. No. PMC1N, Lot 10892, Bangs Laboratory Inc., Fishers, IN, USA) were vortexed and 1 mg of MPIO transferred to a 2 mL plastic tube. 2-(N-morpholino)ethanesulfonic acid (MES) buffer was added (15 mM, 1 mL, pH: 6.0). MPIO were pelleted on a Dynal magnetic separator (Thermo Fisher Scientific, Waltham, MA, USA) for 2 min, and the supernatant discarded. For the amine coupling reaction, 100 μL MES buffer and 100 μL of a 10 mg/mL solution of 1-Ethyl-3-(3-dimethylaminopropyl)carbodiimide (EDC) (Cat. No. E6383, Sigma-Aldrich, St Louis, MO, USA) in 15 mM MES pH 6.0 were added to the pelleted MPIO, and the solution was incubated for 30 min at 25°C on a shaker (1,000 rpm). The solution was washed twice with 1 mL of 15 mM MES buffer and incubated with low endotoxin, 80 μL azide-free monoclonal rat antibody to mouse VCAM-1 (clone M/K-2, Cat. No. 1510-14, SouthernBiotech, Birming-ham, AL, USA) for 24 h at 25°C on a shaker. Conjugates were washed twice with PBS/0.01% Tween and the supernatant containing any unbound antibody discarded. PBS was added to achieve the desired concentration of VCAM-MPIO.

### VCAM-USPIO synthesis

Monocrystalline iron oxide nanocompounds (MION) were synthesized according to the published protocol.^46^ Physicochemical properties of MION have been well investigated previously.^47^ MION were converted into cross-linked iron oxide (CLIO)-NH_2_ by reaction with epichlorohydrin and ammonium hydroxide.^48^ CLIO-NH_2_ were transformed into CLIO-COOH by reaction with succinic anhydride.^30^ For VCAM-USPIO synthesis, MES (0.2 M, 100 μL, pH: 6.0) was added to the solution of CLIO-COOH (1 mg iron, 100 μL, 28.1 nm hydrodynamic diameter, 1.75×10^14^ particles/mg iron) and vortexed. Then, 10 μL of N-hydroxysulfosuccinimide sodium salt (sulfoNHS) (10 μmol; Cat. No. 56485, Sigma Aldrich) in MES buffer (1 M, pH 6.0) and 20 μL of EDC solution (10 μmol; Cat. No. E6383, Sigma Aldrich) in water were added. The mixture was incubated for 15 min at 25°C on a shaker (1,000 rpm), and excess of EDC was quenched by addition of 50 mg of HL-COOH (Tentagel, Cat. No. 86.333, Sigma-Aldrich) for 15 min. The sample was filtered and Tentagel was washed with 100 μL of MES buffer (0.2 M, pH: 6.0). To this solution, 80 μL of anti-mouse VCAM-1 antibody (same as MPIO) was added, and the reaction was incubated for 24 h at 25°C on a shaker. Subsequently, to quench any remaining activated carboxylic acid, 100 μL of borate buffer (1M, pH: 8.5) was added and the solution incubated for another 24 h at 25°C on a shaker. The sample was centrifuged at 186,000 *g* for 1.5 h, the supernatant discarded, and the pellet reconstituted in 1 mL of PBS. The sample was pelleted again (186,000 *g* for 1.5 h), then reconstituted in 1 mL of PBS and pelleted as before. This final pellet was reconstituted in 100 μL of PBS.

### Physicochemical properties of VCAM-MPIO and VCAM-USPIO

The particle size of VCAM-USPIO was measured by dynamic light scattering on a Zetasizer Nano ZS (Malvern Panalytical Ltd, Malvern, UK), and reported as the *z*-average diameter. The polydispersity is reported as the coefficient of variation (CV) of the size distribution, which was calculated as the square root of the mean of the polydispersity index (for USPIO) or provided by the manufacturer (for MPIO). The zeta potential was measured on a Zetasizer Nano ZS in 0.1 M 4-(2-hydroxyethyl)piperazine-1-ethanesulfonic acid buffer at pH 7.0. The particle size of MPIO was provided by the manufacturer as a volume-weighted mean diameter measured with a Coulter counter. The iron content of VCAM-MPIO and VCAM-USPIO was measured by inductively coupled plasma optical emission spectrometry (ICP-OES) on a Varian Vista MPX (Varian, Palo Alto, CA, USA) after acid digestion. The upper limit of COOH loading was estimated from the nitrogen content of a freeze-dried sample, which was measured by combustion analysis. Results are reported as mean ± SD of repeated measurements on the same sample.

### Iron content per particle and estimation of per-particle properties

The mass of iron per particle was used as a conversion factor to relate intrinsic properties relative to the amount of iron (value per mmol of iron) to per particle properties (value for one particle). The mass of iron per particle was calculated as

$$\text{pg}\;\text{Fe/particle}=10^{9}\times \frac{\%\text{Fe}}{100}\times {\left(\text{particles/mg}\right)}^{-1}$$

where %Fe is the weight/weight concentration of iron in a dry sample measured by ICP-OES and particles/mg is the number of particles per mg of dry sample. The latter is provided by the manufacturer for MPIO, while for USPIO it is calculated as

$$\text{Particles}/\text{mg}=10^{-3}\times {\left[\frac{4}{3}\pi {\left(\frac{d}{2}\right)}^{3}(\phi_{{\text{Fe}}_{3}O_{4}}\rho_{{\text{Fe}}_{3}O_{4}}+(1-\phi_{{\text{Fe}}_{3}O_{4}})\rho_{\text{Dex}})\right]}^{-1}$$

where *d* is the particle diameter, $\phi_{{\text{Fe}}_{3}O_{4}}$ is the volume fraction of magnetite in a dry sample, $\rho_{{\text{Fe}}_{3}O_{4}}$ is the density of magnetite, and *ρ*_Dex_=1.07 g/cm^3^ is the density of hydrated cross-linked dextran.^49^ The volume fraction of magnetite was calculated from the mass fraction of magnetite $\chi_{{\text{Fe}}_{3}O_{4}}=(\%\text{Fe}/100)\times [(3A_{r}(\text{Fe})+4A_{r}(O))/(3A_{r}(\text{Fe}))]$, where *A_r_*(Fe)=55.845 u and *A_r_*(O)=15.999 u are the atomic weights of iron and oxygen, respectively, and the density ratio $\rho_{{\text{Fe}}_{3}O_{4}}/\rho_{\text{Dex}}$.

### Assessment of VCAM antibody loading on MPIO and USPIO

MPIO (10 μg) or USPIO (70 μg) were diluted in 200 of PBS buffer, and 2 μL of Alexa Fluor 647 goat anti-rat IgG (H+L) (Cat. No. A21091, 5 fluorophores per antibody, Invitrogen) was added. For MPIO, samples were shaken for 30 min, pelleted using a magnet, washed twice with PBS/0.01% Tween, and resuspended in 400 μL of PBS. Qifikit calibration beads (Dako, Agilent Technologies, Stockport, UK), used as a reference, were prepared according to the manufacturer’s protocol, but substituting the provided fluorescently-conjugated antibody by Alexa Fluor 647 goat anti-mouse IgG (H+L) (Cat. No. A21050, 5 fluorophores per antibody, Invitrogen). Flow cytometry experiments were performed on a BD FACS-Calibur flow cytometer, using channel FL4 (661/16 nm band pass filter). Calibration curve was performed, according to manufacturer protocol, by linear regression of the log–log plot of antibody loading versus fluorescence intensity. For USPIO, sample was centrifuged at 186,000 *g* for 1.5 h, the supernatant discarded, and the pellet reconstituted in 1 mL of PBST. The sample was pelleted again (186,000 *g* for 1.5 h) and reconstituted in 200 μL of PBS. Fluorescence intensity of the samples was measured and compared to a calibration curve composed of serial dilutions of Alexa Fluor 647 goat anti-rat IgG (H+L). Results are reported as mean ± SD.

### Determination of MPIO and USPIO relaxivity in agarose gel

MRI phantoms were prepared as described previously.^30^ Unconjugated MPIO and USPIO were embedded in 2% w/v agarose gels at iron concentrations of 0.0025–0.2 mM. Gels were prepared in 15 mL tubes and heated at 95°C. MPIO and USPIO particles were added to the tubes and dispersed for a homogenous mixture. Samples were sonicated and kept undisturbed at room temperature until cool. MPIO and USPIO relaxitivities were measured using a SE multi-slice sequence in a 7.0 T horizontal bore system (Agilent Technologies, Santa Clara, CA, USA) with a 26-mm birdcage RF coil (Rapid Biomedical, Rimpar, Germany). Sequence parameters for the *T*_2_ maps were: repetition time (TR) =10 s, 12× echo time (TE) =[10–300] ms, spectral width (SW) =100 kHz, matrix =128×128, field of view (FoV) =72×72 mm, slice thickness =2 mm, and experimental time (Texp) =4 h 16 min. Sequence parameters for the *T*_1_ maps were: TR =10 s, TE =10 ms, 12×Ti =[0.01–6.0] s, SW =100 kHz, matrix =128×128, FoV =72×72 mm, and slice thickness =2 mm, Texp =4 h 16 min.

### Assessment of VCAM-MPIO and VCAM-USPIO circulatory half-life

To determine the blood half-life of VCAM-MPIO and VCAM-USPIO, the particles were injected via a tail vein into female BALB/c mice (6–7 weeks old, n=15, Charles River, UK, Kent, UK) at a constant dose of 4 mg Fe/kg in 100 μL sterile PBS. At 0, 0.8, 1, 2, 2.5, 3, 5, and 10 min after injection for VCAM-MPIO and 1, 6, 13, 24, 48 and 72, 96 h after VCAM-USPIO injection, the mice underwent anesthesia with 2%–3% isoflurane in 30% O_2_ with 70% N_2_ followed by thoracotomy. Blood was collected from the left ventricle using a 1 mL syringe with a 23G needle containing 1 μL of heparin sodium (25,000 I.U./mL, Wockhardt Ltd, Wrexham, UK). Blood samples (up to 1 mL) were oxygenated by placing in 15 mL tubes with a 100% O_2_ atmosphere and inverting gently for 5 min until the color changed to bright red. Final blood volume (100 μL) was mixed with 1% agarose solution in PBS (100 μL), at 41°C and placed in 500 μL microcentrifuge tube. *T*_2_ relaxivities of samples were immediately measured using a 7.0 T MRI. For *T*_2_ relaxometery, a simple 90° and 180° pulse sequence were used with long TR (10 s) and adjusted array of TE for best fitting. Circulatory blood half-life was determined from the fitted 1 phase decay curve, constrained to a plateau at the average naive blood sample value (baseline). Blood samples from naïve mice were used as the baseline (n=6).

### Mouse neuroinflammation model

All animal procedures were performed under the University of Oxford guidelines and approved by the UK Home Office. Female BALB/c mice (6–7 weeks old, n=24; Charles River, Kent, UK) were anesthetized with isoflurane (1.5%–2.0%) in 30% O_2_: 70% N_2_O, placed in a stereotaxic frame and a burr hole drilled above the injection site (co-ordinates from bregma: anterior +0.5 mm; left 2.0 mm; depth 2.5 mm). Using a glass microcannula (tip diameter *ca*. <50 μm), 1 ng of mouse recombinant interleukin-1β (IL-1β, Peprotech EC, London, UK) in 1 μL sterile PBS was injected into the left striatum to induce unilateral vascular activation.^25^ The scalp incision was sutured, and the animals were recovered from anesthesia. Three hours after the surgery,^25^ the mice were injected via tail vein with the dose of 4 mg Fe/kg of either (a) VCAM-MPIO (n=12) or (b) VCAM-USPIO (n=12).

### MRI

All MRI experiments were performed using horizontal, wide-bore superconductive 4.7, 7.0, or 9.4 T MRI systems (Agilent Technologies Inc). The same size (ID =26 mm) transmit/receive birdcage RF coils were used in all experiments (Rapid Biomedical).

Mice injected intravenously with VCAM-MPIO (n=4 for each field strength) were imaged 1 h after contrast agent administration, as previously reported. An imaging time-point of 13 h post-injection was chosen for mice injected with VCAM-USPIO (n=4 for each field strength) to account for the longer circulation half-life.^50^,^51^ All mice were imaged with both multi-gradient echo and balanced steady-state free precession 3D sequences (MGE3D and bSSFP3D, respectively). For MGE3D, the parameters were as follows: excitation angle =15°; TR =65.1 ms; time of the 1st echo (TE_1_) =2.5 ms; echo separation time (TE_2_) =4 ms (even echoes acquired in the reverse k-space direction, during opposite read gradient); SW =100 kHz (acquisition time =2.56 ms); and number of echoes =15. For bSSFP3D, the parameters were as follows: excitation angle =20°; TR/TE =8/4 ms; SW =147 kHz (acquisition time =1.74 ms); and number of excitation frequency offsets =8. The following parameters were consistent for both sequences: FoV =22.5×22.5×22.5 mm^3^; acquisition matrix size =256×192×192 (final images zero-filled to 256×256×256; final isotropic resolution =88 μm). Both MGE3D and bSSFP3D scans were run with a single average (NT =1), and the total acquisition time was ~40 min for each.

### Image reconstruction

Images were reconstructed using home-built MATLAB (MathWorks, Natick, MA, USA) code. The final MGE3D images were reconstructed as the square root of sum of squares (SqrtSOS) of individual echoes.^52^–^54^ Based on previous data, second single echo (TE =6.5 ms) was extracted from the MGE3D sequence to represent a single echo gradient echo (GE3D) dataset.^33^,^55^ For bSSFP3D, the final images were reconstructed by adding the individual frequency offsets using the same SqrtSOS algorithm as above.^56^,^57^

### Image and data analysis

For all datasets, brain masks were generated manually. Quantification of hypointense signals from MPIO and USPIO was performed using in-house MATLAB code. Signals arising from ventricles or sinuses, which naturally appear hypointense, were excluded.^27^ The quantification of signal-to-noise (SNR) was performed by measuring the signal intensity (SI_brain_) within the mouse brain (excluding hypointensities) and SD of background noise (S_Dnoise_). For contrast-to-noise (CNR), we additionally measured the signal intensity coming from VCAM-MPIO or VCAM-USPIO (SI_void_). Thus, SNR and CNR were estimated using the following calculations, respectively:

$$\text{SNR}=\frac{{\text{SI}}_{\text{brain}}}{{\text{SD}}_{\text{noise}}}$$

$$\text{CNR}=\frac{{\text{SI}}_{\text{brain}}-{\text{SI}}_{\text{void}}}{{\text{SD}}_{\text{noise}}}$$

Statistical analyses were performed using Prism software (GraphPad, San Diego, CA, USA). The normality of the measured variables was tested using the Shapiro–Wilk test. For multiple comparisons, analysis of variance (ANOVA) followed by Tukey post-hoc test were used.

### Ex vivo assessment of VCAM-MPIO and USPIO binding

Following imaging, mice were transcardially perfused under terminal anesthesia with 0.9% heparinized saline followed by periodate-lysine-paraformaldehyde (PLP-light) containing 0.1% glutaraldehyde. Brains were harvested, post-fixed in PLP then placed in 30% sucrose solution until they sank to the bottom. Samples were frozen in isopentane at −80°C. Brains were cut at 10 μm and kept at −20°C until immunohistochemi-cal staining for co-localization of VCAM-1 and iron oxide particles. Sections were quenched with 1% (v/v) hydrogen peroxide (30% w/v) in methanol (Sigma Aldrich), blocked with 10% goat serum in PBS for 1 h, and then incubated overnight at 4°C with the primary VCAM-1 antibody (1:100; SouthernBiotech). Sections were washed using PBS/0.01% Tween (Sigma Aldrich), and then incubated with a biotinylated rat polyclonal secondary antibody (1:200 Abcam, Cambridge, UK) for 1 h. Slides were washed and then incubated in Vectorelite ABC kit (1:1:100; Vector Laboratories, Peterborough, UK) for 45 min. The peroxidase was visualized using 3,3′-diaminobenzidine (Sigma Aldrich). To detect the iron oxide particles, sections were stained using Perls’ Prussian blue. Positive control liver and spleen samples from mice injected intravenously with USPIO were included for Perls’ Prussian blue staining. For fluorescent VCAM-1 staining, Tris-NaCl blocking buffer (TNB; PerkinElmer, Seer Green, UK) was used. Sections were subsequently incubated overnight at 4°C with the VCAM-1 primary antibody, as above. Sections were washed with PBS and incubated with biotinylated anti-goat secondary in TNB for 30 min. Sections were then washed with PBS, incubated with streptavidin–horseradish peroxidase (1:200; PerkinElmer) in TNB for 30 min, washed and incubated for 7 min in the dark with tyramide signal amplification (TSA)-biotin (1:100) in amplification buffer (PerkinElmer). Slides were washed and incubated with a streptavidin-DyLight 488 (1:200; Vector Laboratories). Samples were coverslipped, using Vectashield mounting medium containing DAPI (Vector Laboratories). Whole brain fluorescent images were taken at 20× magnification using an Aperio immunofluorescence slide scanner (Leica Biosystems, Milton Keynes, UK).

## Results

### Physicochemical properties of VCAM-MPIO and VCAM-USPIO

The *z*-average hydrodynamic diameter of VCAM-USPIO was 28.1 nm. The particle size distribution (Figure S1) is moderately polydisperse, with a CV of 36%. The particle size distribution of MPIO was narrower, with a CV of 15% and a mean diameter of 880 nm. Both USPIO and MPIO were negatively charged, with a zeta potential of −9.3±0.9 mV and −25.6±1.3 mV, respectively. The iron content of USPIO and MPIO was 30.6% w/w and 27.8% w/w, respectively, as measured by ICP-OES.

Quantitation of antibody loading showed the density of VCAM antibody to be 10,490±450 molecules per particle (n=4) on MPIO and 7.4±3.1 molecules per particle on USPIO (n=3). Although the number of antibodies per particle differs by 3 orders of magnitude, the surface density (number of antibodies per unit area) is comparable, with 4,320±200 molecules/μm^2^ on MPIO and 3,000±1,300 molecules/μm^2^ on USPIO. The zeta potential of either agents does not change significantly after conjugation to the antibody (−10.3±1.1 mV for VCAM-USPIO and −26.4±1.4 mV for VCAM-MPIO), which is not entirely unexpected as the measurement pH falls within the isoelectric point range of rat IgG.^58^

The *R*_2_ relaxivity of MPIO and USPIO, measured in agarose phantoms, was found to be 500 Hz L/mmol Fe and 126 Hz L/mmol Fe, respectively (Figure S2A and B). Both values are consistent with previous literature reports^30^,^37^,^59^,^60^ and correspond to average relaxivitiy per particle of 1.18×10^−9^ Hz L/particle for MPIO and the much lower value of 1.29×10^−14^ Hz L/particle for USPIO. Properties of MPIO and USPIO are summarized in Table 1.

### Comparison of MRI sequences and field strengths for detection of iron oxide particles

**Figure 1:**
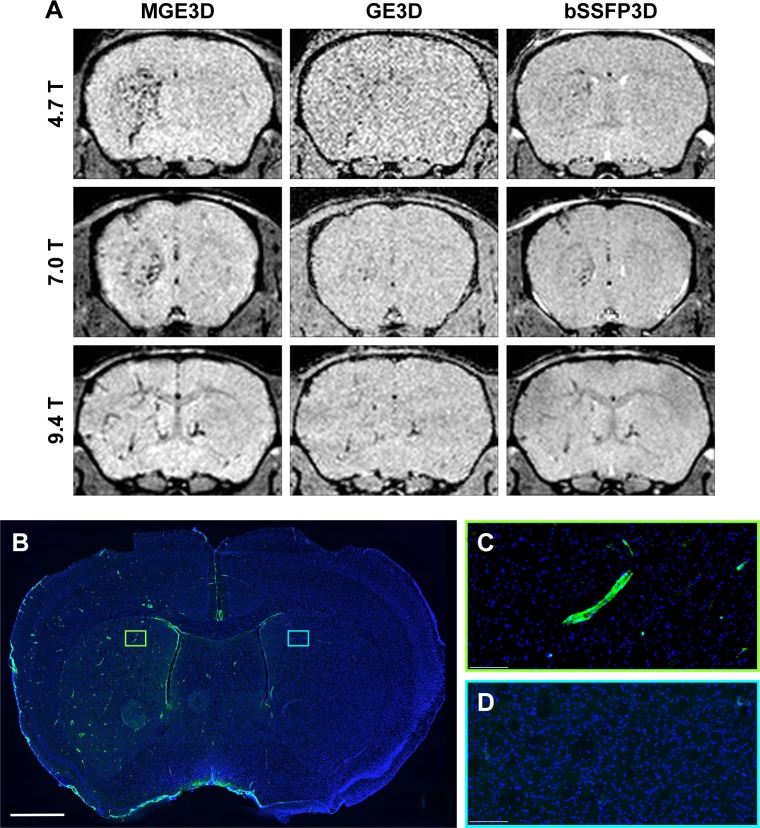
(**A**) Representative in vivo images of mice 1 h post-VCAM-MPIO injection at different magnetic fields. Three sequences of MGE3D, GE3D, and bSSFP3D are shown from a 3D dataset. Intense low signal areas on the left side of the brain reflect the specific retention of MPIO on acutely activated vascular endothelium with visually absent contrast effect in the contralateral control hemisphere. MGE3D demonstrates better visualization of hypointensities compared with other sequences. (**B**–**D**) Histological assessment of VCAM upregulation at the injection side. **Notes:** Green vessels show unilateral upregulation of VCAM, nuclei are shown in blue. Scale bar: 1 mm. **Abbreviations:** bSSFP3D, balanced steady-state free precession 3D; GE3D, gradient echo 3D; MGE3D, multi-gradient echo 3D; MPIO, microparticles of iron oxide; VCAM, vascular cell adhesion molecule.

**Figure 2:**
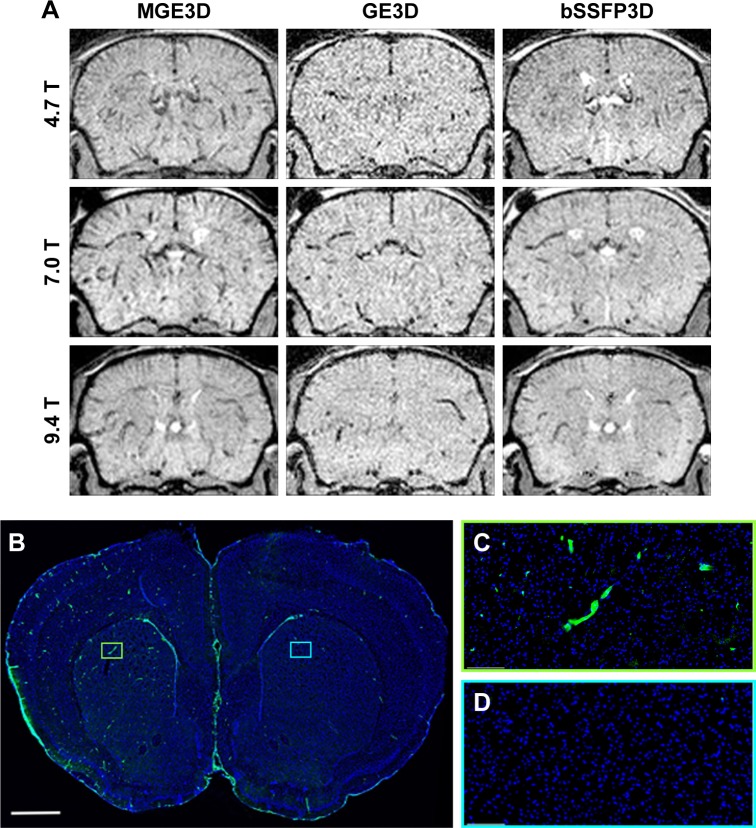
(**A**) Representative in vivo images of mice 13 h post-VCAM-USPIO injection at different magnetic fields. Three sequences of MGE3D, GE3D, and bSSFP3D are shown from a 3D dataset. Hypointensities were detected in both hemispheres of the brains. (**B**–**D**) Histological assessment of VCAM upregulation at the injection side. **Notes:** Green vessels show unilateral upregulation of VCAM, nuclei are shown in blue. Scale bar: 1 mm. **Abbreviations:** bSSFP3D, balanced steady-state free precession 3D; GE3D, gradient echo 3D; MGE3D, multi-gradient echo 3D; USPIO, ultra-small superparamagnetic iron oxide; VCAM, vascular cell adhesion molecule.

**Figure 3:**
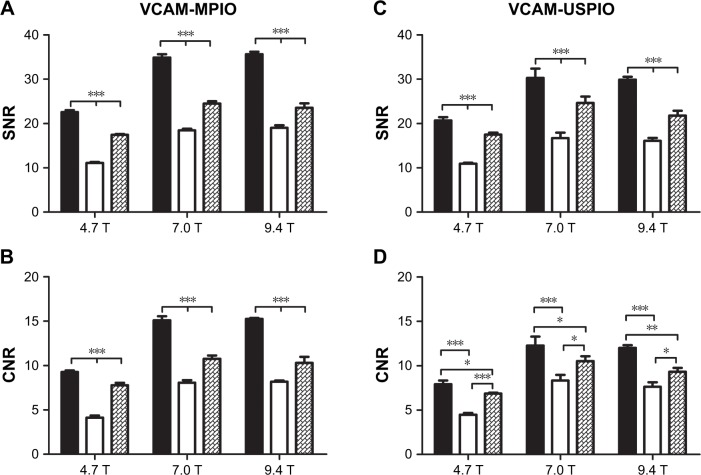
Quantitative comparison of MGE3D (■), GE3D (□), and bSSFP3D ([img]) sequences for detection of MPIO and USPIO at different magnetic field strengths. **Note:** MGE3D yielded significantly higher SNR (**A**, **C**) and CNR (**B**, **D**) compared with other sequences at all field strengths for both MPIO and USPIO detection (*P<0.05, **P<0.01, ***P<0.001). **Abbreviations:** bSSFP3D, balanced steady-state free precession 3D; CNR, contrast-to-noise ratio; GE3D, gradient echo 3D; MGE3D, multi-gradient echo 3D; MPIO, microparticles of iron oxide; SNR, signal-to-noise ratio; USPIO, ultra-small superparamagnetic iron oxide; VCAM, vascular cell adhesion molecule.

Representative images obtained using each of the 3 sequences (MGE3D, GE3D, and bSSFP3D) at 4.7, 7.0, and 9.4 T, with the same resolution and scan time, are shown in Figures 1A and 2A. Qualitatively, all 3 sequences enabled visualization of hypointense foci in the brains of mice injected with either VCAM-MPIO (Figure 1A) or VCAM-USPIO (Figure 2A). However, the *T*_2_*-weighted MGE3D sequence appeared to reveal hypointensities more prominently than either the *T*_2_*-weighted GE3D or the *T*_2_/*T*_1_-weighted bSSFP3D sequence. Quantitative assessment of SNR (Figure 3A and C) and CNR (Figure 3B and D) for all sequences at each magnetic field confirmed that the highest SNR was conferred by the MGE3D sequence compared with both the other sequences for both MPIO and USPIO (in all cases, ANOVA – *P*<0.0001, followed by Tukey’s post-hoc test; Figure 3). The *T*_2_/*T*_1_-weighted bSSFP3D sequence also showed significantly greater CNR than the standard GE3D sequence for the USPIO (Figure 3D). When comparing SNR and CNR of the optimal MGE3D sequence at different magnetic field strengths, a significant increase in both SNR and CNR was evident from 4.7 to 7.0 T (in all cases, ANOVA – *P*<0.05, followed by Tukey’s post-hoc test). However, no additional increase in either signal or contrast was gained by increasing field strength from 7.0 to 9.4 T.

### Comparing sensitivity and specificity of VCAM-MPIO and VCAM-USPIO

**Figure 4:**
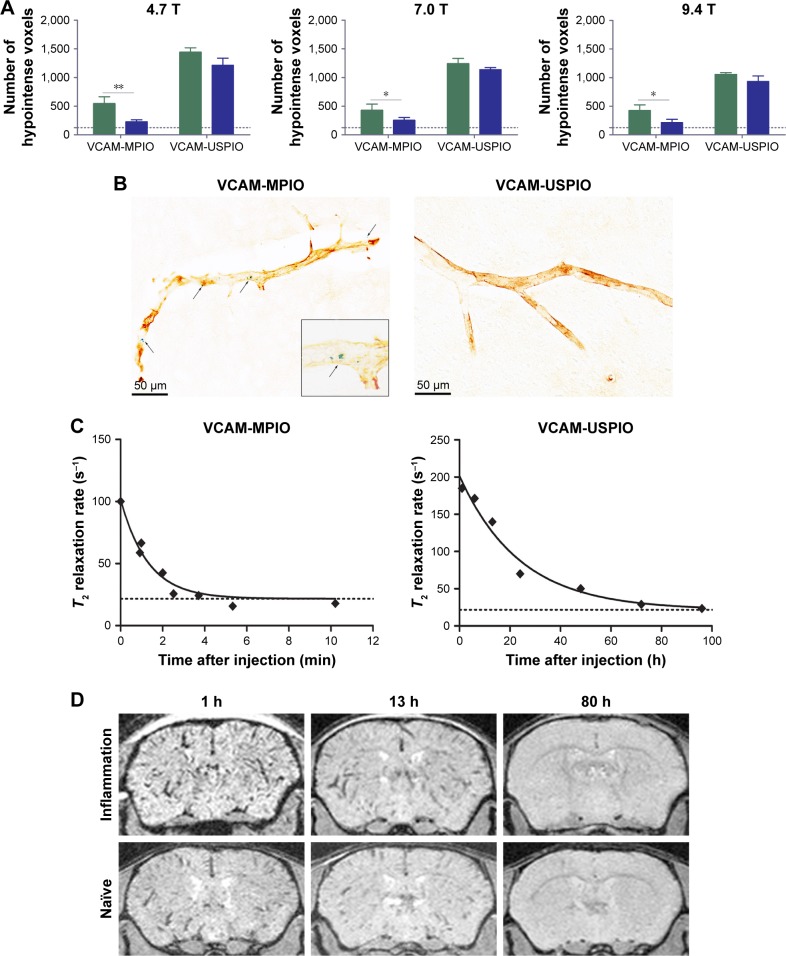
(**A**) Graph showing quantitation of MPIO- and USPIO-induced hypointensities in injected (green bar) and contralateral (blue bar) hemispheres using MGE3D sequence. Baseline level of endogenous hypointensities is shown with the dotted line. A significant difference was evident between the 2 hemispheres for VCAM-MPIO-injected mice at all field strengths. No significant difference was found for VCAM-USPIO. N=4 per group; *P<0.05, **P<0.01. (**B**) Histological staining for VCAM-1 (brown) and iron beads (Perls’ Prussian blue) demonstrating upregulation of VCAM-1 on endothelial cells together with VCAM-MPIO binding (arrows). VCAM-USPIO was not detected in brain samples after perfusion, error bar=50 um (**C**) Graph showing the decay curve of the biological clearance of VCAM-MPIO and VCAM-USPIO, at a constant dose of 4 mg Fe/kg of body weight over time after injection. Graph shows *T*_2_ relaxivity values of blood samples at different time points after administration. Curve is fitted as a single-phase exponential decay constrained to a plateau at the average naïve blood sample values (baseline; dotted lines). (**D**) MGE3D images of mice injected with VCAM-USPIO at different time-points acquired at 7.0 T – images show the presence of VCAM-USPIO in an inflammation model (top row) and naïve mouse (bottom row) at 1 and 13 h after contrast agent administration. No signal was detected 80 h after injection in both cohorts. **Abbreviations:** MGE3D, multi-gradient echo 3D; MPIO, microparticles of iron oxide; USPIO, ultra-small superparamagnetic iron oxide; VCAM, vascular cell adhesion molecule.

Immunohistochemically, unilateral upregulation of VCAM-1 was evident in mice injected intracerebrally with IL-1β and intravenously with VCAM-MPIO (Figure 1B–D). In accord with this unilateral upregulation, a marked unilateral contrast effect was evident in brain images at all field strengths and with all 3 sequences (Figure 1A). In contrast, in mice injected intracerebrally with IL-1β and intravenously with VCAM-USPIO, bilateral contrast effects were evident in all animals, with no differences between the hemispheres (Figure 2A). Immunohistochemistry confirmed unilateral VCAM-1 upregulation (Figure 2B–D). Quantitative analysis of hypointense voxels (mean ± SE) for all 3 field strengths showed a significant difference between the number of hypointense voxels in the injected and contralateral hemispheres for mice injected with VCAM-MPIO (4.7 T: 446±119 vs 229±30; 7.0 T: 430±47 vs 253±47; 9.4 T: 425±98 vs 214±58; n=4 per group, *P*<0.05; Figure 4A), but no significant difference for VCAM-USPIO-induced hypointensities (4.7 T: 1,444±75 vs 1,213±125; 7.0 T: 1,244±90 vs 1,138±36; 9.4 T: 1,058±28 vs 933±95; n=4 per group). Immunohistochemically, VCAM-1 and Perls’ Prussian blue staining revealed the presence of VCAM-MPIO in the lumen of VCAM-1-positive vessels following MRI. However, despite intense bilateral contrast effects observed by MRI, no VCAM-USPIO were detected on VCAM-1 positive vessels or within the brain parenchyma following MRI (Figure 4B). To test that Perls’ Prussian blue stain can, in fact, stain and detect USPIO, positive control samples (liver and spleen) from mice injected with USPIO were stained. The results indicate that it is possible to detect USPIO (Figure S3) histologically.^61^

To determine whether contrast effects could be arising from unbound VCAM-USPIO within the blood, the half-life of VCAM-USPIO in the circulation was measured and found to be 16.5 h, in comparison to 1.2 min for VCAM-MPIO (Figure 4C). Consequently, the bilateral contrast effects arising from VCAM-USPIO administration was further investigated in both naïve mice and mice injected intracerebrally with IL-1β. Mice were injected intravenously with VCAM-USPIO and imaged at different time points (1, 13, and 80 h) after injection, as above (Figure 4D). Results showed bilateral contrast effects and, hence, presence of VCAM-USPIO, in both cohorts at 1 and 13 h after injection. In both naïve and IL-1β injected mice, contrast effects arising from VCAM-USPIO were no longer evident 80 h after injection (ie, *ca*. 5× blood half-life of VCAM-USPIO).

## Discussion

In this study, we have demonstrated that the *T*_2_*-weighted multi-gradient echo sequence provides the highest SNR and CNR for detection of targeted iron oxide particles in mouse brain. All sequences showed an improvement in both SNR and CNR on increasing field strength from 4.7 to 7.0 T, but no further improvements in these parameters were observed on going to higher field. Finally, we have shown that while targeted MPIO enable selective and specific detection of target expression on the cerebral vasculature, the long half-life and lower relaxivity, and hence CNR, per particle of the targeted USPIO will likely render these particles impractical for most applications of molecular MRI targeting endovascular ligands.

Several different MRI approaches have been used previously for mouse neuroimaging of iron oxide contrast effects. As the transverse magnetization decays with *T*_2_*, it distorts the gradient echo and, therefore, induces geometrical distortions.^62^ Hence, conventional *T*_2_*-weighted gradient echo (GE3D) is the most commonly used sequence for imaging of these particles.^36^ This sequence uses a single echo sampled per line in k-space. In contrast, for *T*_2_*-weighted multi-echo gradient echo (MGE3D), the recovery time is used to acquire additional echoes without increasing the total experimental time. Thus, a series of echoes at different TE are recorded for each line in k-space. This additional information can be used in a number of different ways, such as applying a SqrtSOS algorithm on the magnitude images from each echo to obtain MGE3D images.^63^ The bSSFP3D sequence also has been used previously for tracking cells labeled with iron oxide particles and has shown advantages over conventional *T*_2_- and *T*_2_*-weighted sequences.^29^,^41^,^64^ However, this sequence is highly sensitive to local field inhomogeneities and the resulting “banding artifact” worsens at higher magnetic fields.^65^,^66^

The results of the current study suggest that by combining echoes in the MGE3D dataset, both SNR and CNR show significant improvements. Thus, MGE3D provides better depiction of both VCAM-MPIO and VCAM-USPIO contrast effects and greater overall image quality and sharpness compared to conventional GE3D and bSSFP3D. Consequently, quantitation of contrast effects will be improved significantly using the MGE3D sequence.

With regard to field strength, theoretically, an improvement in SNR would be anticipated with increasing field strength. However, many factors can limit the results observed in practice. In this study, improvements in both SNR and CNR were achieved for all sequences between 4.7 and 7.0/9.4 T, in accord with other rodent neuroimaging studies.^67^,^68^ Although a significant improvement was observed between 4.7 and 7.0 T, no significant gain was obtained by increasing field strength further to 9.4 T in the current study. One possible reason for this lack of further SNR benefit is that magnetic susceptibility inherent in a mouse brain produce field-dependent perturbations in B_0_ homogeneity, which result in degradation of SNR at 9.4 T. However, direct comparison of different magnetic field strengths suffers from several practical limitations.^67^ For example, one consideration is the effect of different bore sizes. For the systems used in this study, the 9.4 T scanner had the smallest bore size (ID 160 mm), only allowing the use of a gradient coil with lower performance (gradient coil dimensions: OD 156 mm, ID 100 mm) than the 210 mm bore 7.0 T and 310 mm bore 4.7 T systems (gradient coil dimensions in both cases: OD 205 mm, ID 120 mm). Although it is not possible to generalize these results to all MRI scanners, given the vast array of different set-ups, our findings suggest that improved detection of iron oxide particles will be obtained by working at 7.0 T compared to lower field strengths. Beyond this field strength, the negative impact of field inhomogeneities and system constraints may counteract the benefits of higher field.

It has been shown that in response to disease, injury, and inflammation, VCAM-1 upregulates rapidly on activated endothelial cells of the brain.^69^ VCAM-1-targeted MPIO have been widely studied by a number of groups for detection of VCAM-1 in preclinical models of disease and have shown high sensitivity and specificity for detecting this biomarker in vivo.^25^,^33^–^35^,^37^,^70^–^73^ As a simple model for inducing endothelial activation, we have previously demonstrated that a focal unilateral injection of IL-1β causes unilateral hemispheric upregulation of adhesion molecules on the endovascular surface, including VCAM-1.^25^,^70^ We, therefore, used this model to determine the relative sensitivity and specificity of VCAM-MPIO and VCAM-USPIO for detection of target expression in vivo and found dramatically different results. For VCAM-MPIO, a combination of high valency targeted delivery of a high payload of iron oxide to sites of inflammation, and rapid clearance of MPIO from the blood conferred a high degree of specificity and sensitivity for target detection, as reported previously.^25^,^30^,^33^,^39^ These properties are crucial in detecting biomarkers such as VCAM-1, that have relatively low abundance on endothelial cells^74^ and may only be detectable using particles with higher contrast owing to their greater iron payload.

In contrast to VCAM-MPIO, no target-specific binding was detected by either MRI or histology (using Perls’ Prussian blue staining) in mice injected with VCAM-USPIO. Moreover, our data show that VCAM-USPIO were still present in the blood circulation 13 h post-administration, in both naïve and IL-1β injected mice. As a consequence of this long circulation time, marked background contrast effects were evident, which preclude detection of target-specific binding in the injected hemisphere. This is not to say that target-specific binding is entirely absent in the case of VCAM-USPIO, but rather that it is undetectable owing to the continuing presence of circulating agent and the low relaxivity, and hence CNR, of the particles that are bound. Although, theoretically, it may be possible to identify an imaging window at which point all circulating USPIO have been eliminated, it is unlikely that specific binding will still be present and detectable at that time point. Moreover, the necessity for a long time window between contrast administration and imaging would also be less favorable for downstream clinical use, than an agent that allowed administration and rapid imaging within a short time-frame thereafter. In practice, we were unable to detect any VCAM-USPIO binding on the activated endothelium 13 h after injection histologically, and no specific VCAM-USPIO contrast effects were visible by MRI at 80 h after injection when the blood pool has cleared.

These findings demonstrate empirically that MPIO provide significant benefits for molecularly targeted MRI in brain and exhibit higher sensitivity than USPIO. While for USPIO many effective applications have been reported, the small size of these particles is likely to be limiting for their application in molecular MRI studies, particularly of endovascular targets. One of the drawbacks of MPIO that is often discussed, is their limited translational capacity to the clinic. However, with the new generation of degradable biocompatible particles,^30^ this platform now has realizable translational potential.

## Conclusion

The overall goal of this work was to identify the optimal pulse sequence, field strength, and particle size for molecular MRI in mice. Our results demonstrate clear advantages of the MGE3D sequence over bSSFP3D and GE3D for detection of iron oxide particles in vivo. Further, the specificity and sensitivity of VCAM-MPIO, compared with VCAM-USPIO, for delineation of activated endothelium in a mouse model of acute inflammation has been shown. With advances in the generation of biodegradable micrometer-sized iron oxide particles, these agents look promising for clinical translation in a broad range of central nervous system diseases, including inflammation and cancer.

## Supplementary materials

Figure S1 Particle size distribution (intensity-weighted) of VCAM-USPIO measured by DLS.
**Note:** The 3 traces are repeated measurements of the same sample.
**Abbreviations:** DLS, dynamic light scattering; USPIO, ultra-small superparamagnetic iron oxide; VCAM, vascular cell adhesion molecule.

Figure S2 (**A**) MPIO (top row) and USPIO (bottom row) were embedded in 2% agarose gel at the same iron concentration (triplicates). (A–C): 0.2 mM, (D–F): 0.1 mM, (G–I): 0.05 mM, (J–L): 0.024 mM, and (M–O): PBS. *T*_2_ (ms) and *R*_2_ (Hz) maps generated at 7.0 T are shown. (**B**) *T*_2_ relaxivity was measured for both particles; MPIO showed significantly greater relaxivity (steeper slope; *P*<0.0001) than USPIO. Errors are expressed as mean ± SD for n=3.
**Abbreviations:** MPIO, microparticles of iron oxide; PBS, phosphate buffered saline; USPIO, ultra-small superparamagnetic iron oxide.

Figure S3 Positive control Prussian blue staining for USPIO and counterstaining with nuclear fast red.
**Notes:** (**A**) Mouse liver section, blue: USPIO; pink: nuclei, (**B**) Mouse spleen section, blue: USPIO; pink: nuclei.
**Abbreviation:** USPIO, ultra-small superparamagnetic iron oxide.

## Figures and Tables

**Table 1:** Physicochemical properties of MPIO and USPIO

Properties	MPIO	USPIO
Mean diameter (nm)	880^a^	28.1
Coefficient of variation^b^ (%)	15^a^	36
Iron content (% w/w)	27.8	30.6
Core diameter (nm)	5.1^c^	9–14^d^
Saturation magnetization (emu/g Fe)	115^c^	62^e^
Zeta potential (mV)	−26.4±1.4^f^	−10.3±1.1^g^
COOH loading (μmol/mg Fe)	122^a^	<1.24
Surface area of 1 particle^e^ (μm^2^)	2.4	0.0025
Iron content per particle (pg Fe/particle)	0.13	5.7×10^−6^
Antibody density per particle (Ab/particle)	10,490±450^g^	7.4±3.1^g^
Antibody density per unit area (Ab/μm^2^)	4,320±200^g^	3,000±1,300^g^
Bulk relaxivity (Hz L/mmol Fe)	500	126
Particle relaxivity (Hz L/particle)	1.18×10^−9^	1.29×10^−14^

**Notes:**

a Data provided by the manufacturer.

b Relative SD of particle diameters within a sample.

c From the study by Delangre et al.^59^

d From the study by Perez-Balderas et al.^30^

e From the study by Chen et al.^58^

F Calculated from diameter assuming spherical particles.

g Data are reported as mean ± SD of repeated measurements of the same sample.

**Abbreviations:** MPIO, microparticles of iron oxide; USPIO, ultra-small super-paramagnetic iron oxide.
