# Supplementary figures and images for: Optimization of molecularly targeted MRI in the brain: empirical comparison of sequences and particles
Source: Int J Nanomedicine. 2018 Jul 25;13:4345–59. doi: 10.2147/IJN.S158071 (PMC6064157; doi:10.2147/IJN.S158071)

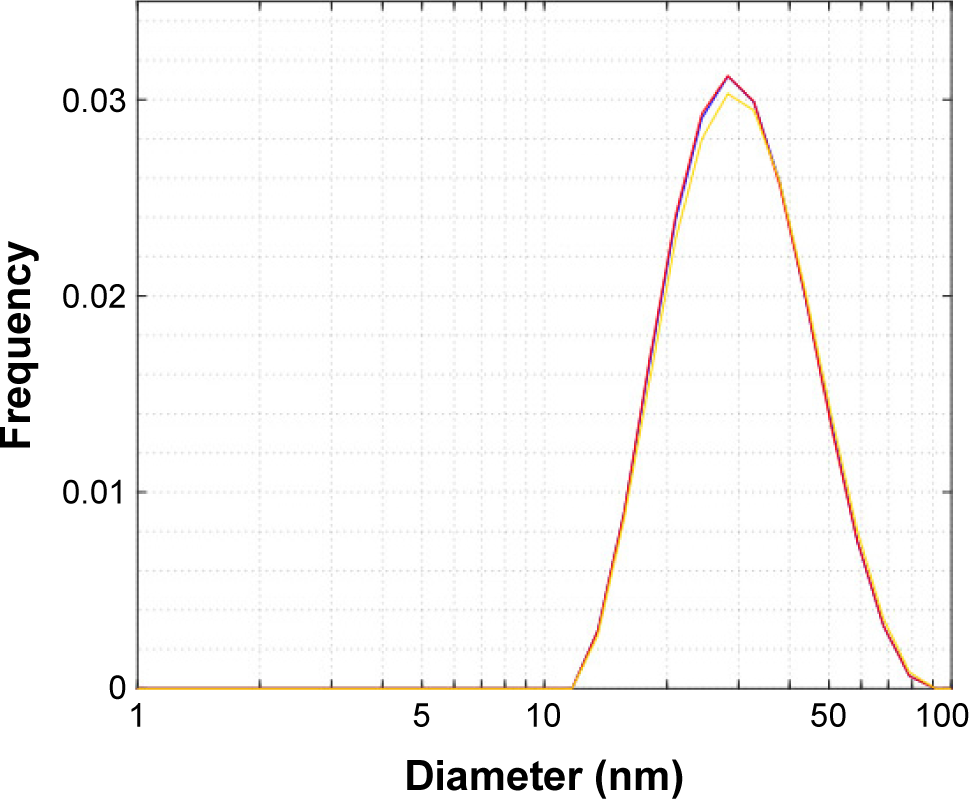

Supplement: Figure S1 — Particle size distribution (intensity-weighted) of VCAM-USPIO measured by DLS. Note: The 3 traces are repeated measurements of the same sample. Abbreviations: DLS, dynamic light scattering; USPIO, ultra-small superparamagnetic iron oxide; VCAM, vascular cell adhesion molecule. [file ijn-13-4345s1.tif]

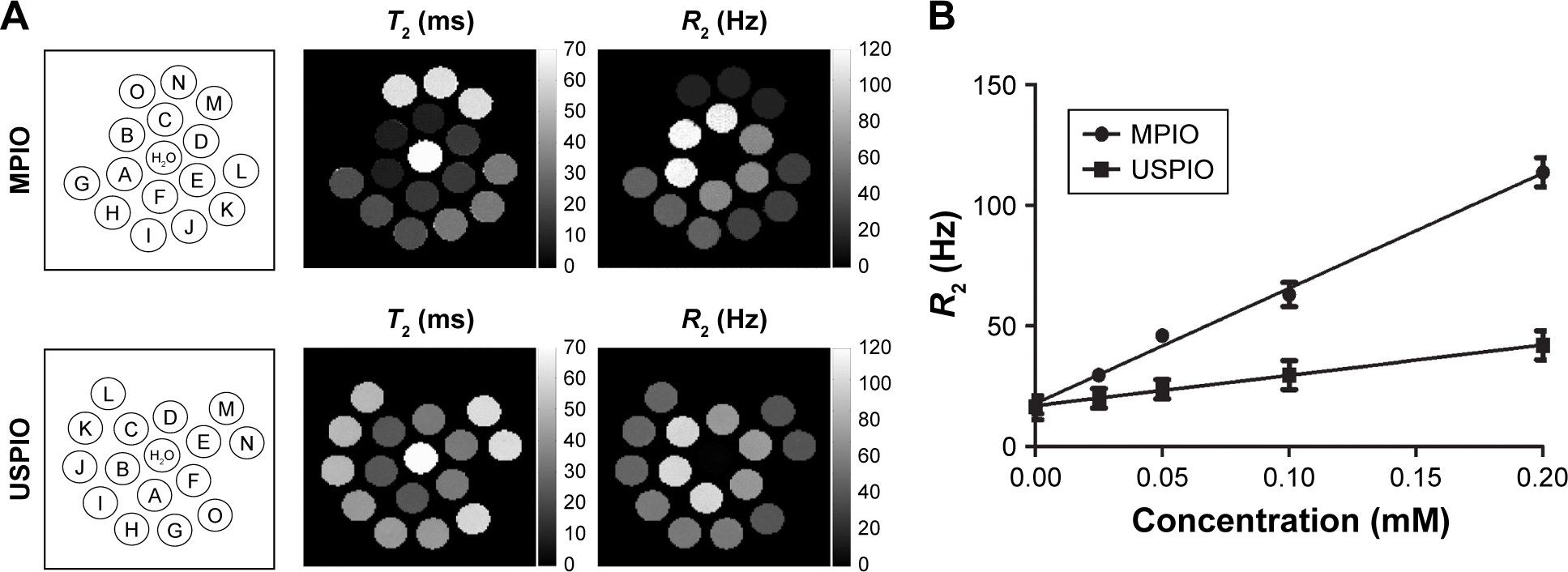

Supplement: Figure S2 — (A) MPIO (top row) and USPIO (bottom row) were embedded in 2% agarose gel at the same iron concentration (triplicates). (A–C): 0.2 mM, (D–F): 0.1 mM, (G–I): 0.05 mM, (J–L): 0.024 mM, and (M–O): PBS. T2 (ms) and R2 (Hz) maps generated at 7.0 T are shown. (B) T2 relaxivity was measured for both particles; MPIO showed significantly greater relaxivity (steeper slope; P<0.0001) than USPIO. Errors are expressed as mean ± SD for n=3. Abbreviations: MPIO, microparticles of iron oxide; PBS, phosphate buffered saline; USPIO, ultra-small superparamagnetic iron oxide. [file ijn-13-4345s2.tif]

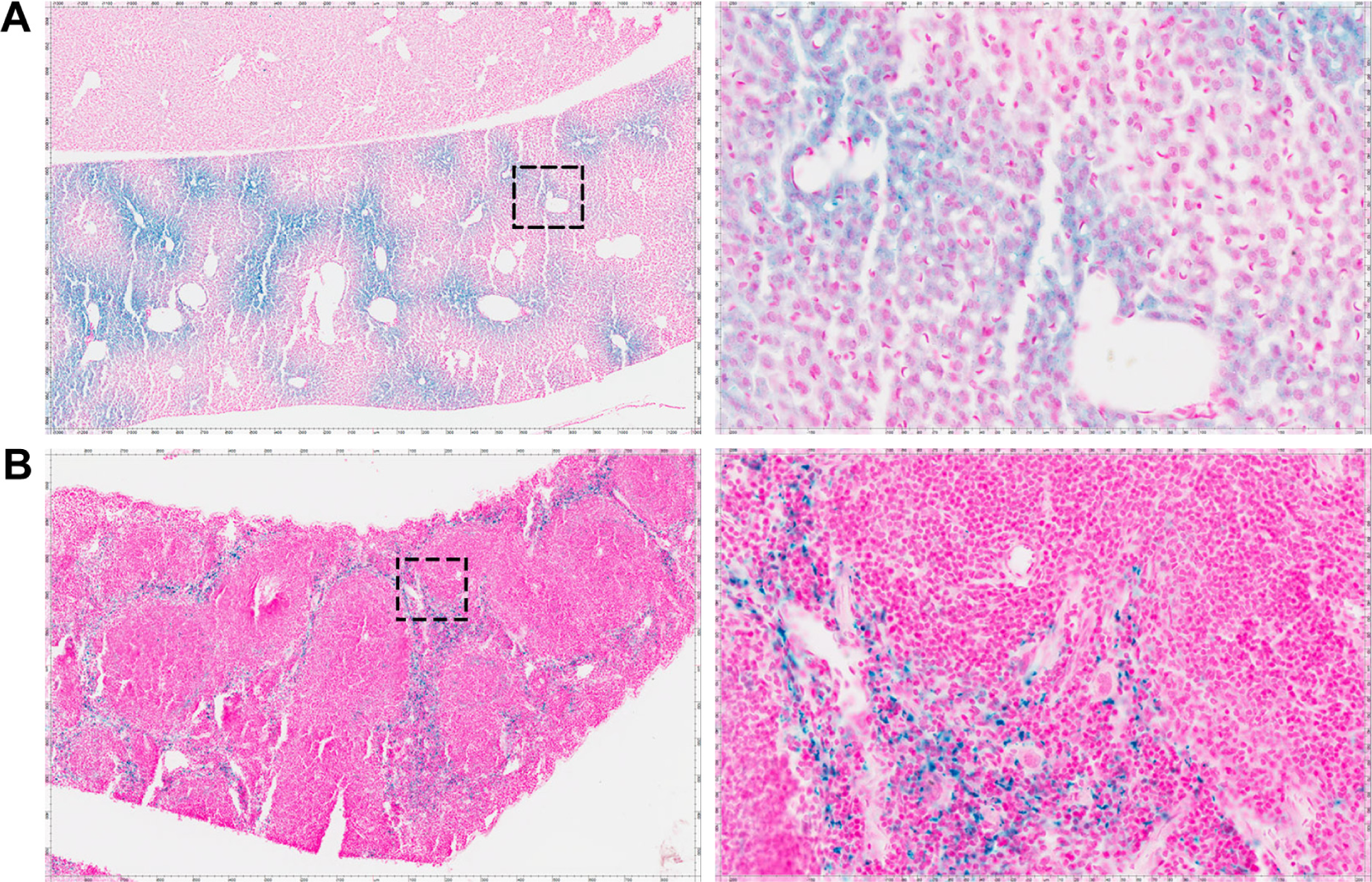

Supplement: Figure S3 — Positive control Prussian blue staining for USPIO and counterstaining with nuclear fast red. Notes: (A) Mouse liver section, blue: USPIO; pink: nuclei, (B) Mouse spleen section, blue: USPIO; pink: nuclei. Abbreviation: USPIO, ultra-small superparamagnetic iron oxide. [file ijn-13-4345s3.tif]
